# The expression of the Slit-Robo signal in the retina of diabetic rats and the vitreous or fibrovascular retinal membranes of patients with proliferative diabetic retinopathy

**DOI:** 10.1371/journal.pone.0185795

**Published:** 2017-10-03

**Authors:** Weiyan Zhou, Hongya Wang, Wenzhen Yu, Wankun Xie, Min Zhao, Lvzhen Huang, Xiaoxin Li

**Affiliations:** 1 Department of Ophthalmology, Shandong Provincial Hospital affiliated to Shandong University, Jinan, China; 2 Department of Ophthalmology, Peking University People’s Hospital, Beijing Key Laboratory of Diagnosis and Therapy of Retinal and Choroid Diseases, Beijing, China; 3 Department of Clinical Laboratory, Shandong Provincial Hospital affiliated to Shandong University, Jinan, China; Indiana University School of Medicine, UNITED STATES

## Abstract

**Purpose:**

The Slit-Robo signal has an important role in vasculogenesis and angiogenesis. Our study examined the expression of Slit2 and its receptor, Robo1, in a rat model of streptozotocin-induced diabetes and in patients with proliferative diabetic retinopathy.

**Methods:**

Diabetes was induced in male Sprague-Dawley rats via a single, intraperitoneal injection of streptozotocin. The rats were sacrificed 1, 3 or 6 months after the injection. The expression of Slit2 and Robo1 in retinal tissue was measured by real-time reverse transcription polymerase chain reaction (RT-PCR), and protein levels were measured by western blotting and immunohistochemistry. Recombinant N-Slit2 protein was used to study the effects of Slit2 on the expression of VEGF in vivo. The concentration of Slit2 protein in human eyes was measured by enzyme-linked immunosorbent assay in 27 eyes with proliferative diabetic retinopathy and 28 eyes in control group. The expression of Slit2, Robo1 and VEGF in the excised human fibrovascular membranes was examined by fluorescence immunostaining and semi-quantitative RT-PCR.

**Results:**

The expression of Slit2 and Robo1 in the retina was altered after STZ injection. Recombinant N-Slit2 protein did not increase the retinal VEGF expression. Vitreous concentrations of Slit2 were significantly higher in the study group than in the control group. In the human fibrovascular membranes of the study group, the co-localization of VEGF with the markers for Slit2 and Robo1was observed. The expression of Slit2 mRNA, Robo1 mRNA, and VEGF mRNA was significantly higher in human fibrovascular proliferative diabetic retinopathy membranes than in the control membranes.

**Conclusions:**

The alteration of Slit2 and Robo1 expression in the retinas of diabetic rats and patients with proliferative diabetic retinopathy suggests a role for the Slit-Robo signal in the various stages diabetic retinopathy. Further studies should address the possible involvement of the Slit-Robo signal in the pathophysiological progress of diabetic retinopathy.

## Introduction

Worldwide, diabetic retinopathy (DR) is the leading cause of blindness among those 20 to 60 years old [1]. Clinically, diabetic retinopathy can be divided into two stages: background and proliferative retinopathy. The clinical features of background diabetic retinopathy include the breakdown of the blood–retinal barrier and various visual deficits[2, 3]. The second stage of diabetic retinopathy leads to visual impairment through bleeding or retinal detachment by accompanying fibrous tissue[4]. In this stage, the expression of angiogenic and anti-angiogenic factors is altered in the vitreous and proliferative vitreoretinopathy membranes. Currently, no established animal models of human diabetic retinopathy are available, although similarities between model organisms and humans exist at the molecular level[5]. These molecular similarities support the use of diabetic rats as an acceptable in vivo model of retinopathy.

The direct cause of DR remains unknown. DR ultimately leads to the proliferative changes associated with neovascularization, which is controlled by a balance of growth factors and cytokines. The angiogenesis factors included vascular endothelial growth factor (VEGF), bFGF, TGF-β, SDF-1 and so on. There is increasing evidence that the migration and patterning of axons and blood vessels share similar guidance mechanisms[6]. The major classes of axon guidance molecules include the netrins, semaphorins, ephrins and Slit ligands, which have a role in repelling guidance cues in the nervous system. Each family has at least one member that plays a functional role in vascular development[7]. In our previous study, We indicated that Robo1, 4 and slit2 may play a role in the formation of fibrovascular membranes (FVMs). They may also be involved in retinal vasculogenesis and angiogenesis. Slit-Robo interactions have recently been shown to function in tumor angiogenesis and to act as attractants during endothelial cell migration[8]. So our this study focuses on the Slit and Roundabout (Robo) family.

In vertebrates, three Slit homologs are known (Slit1, Slit2, Slit3). The Robo family of transmembrane receptors encompasses four members in vertebrates (Robo1, Robo2, Robo3/Rig-1, Robo4/Magic Roundabout). Slits are large secreted proteins that bind to Robo transmembrane receptors and induce a variety of functions[9]. Slit2 has been thought to be the vascular-specific Slit and has been studied extensively[10–12]. However, recent studies of the Slit2 gene have demonstrated a conserved role in regulating the development of the vascular system. For example, some groups report that Slit2 inhibits neovascularization [13, 14, 15]and other groups report that Slit2 acts as a positive stimulus on endothelial cells[16]. Robo1 has also been functionally implicated in the vasculature and in the context of tumor growth. Slits binds directly to it and has been shown to promote endothelial cell motility[17]. However, to date the role of action of Robo1 in endothelial cells remains unresolved[18].

Although there have been many studies examining the Slit-Robo signal, its role in diabetic retinopathy has not yet been demonstrated. The aim of our study was to assess the role of the Slit-Robo signal in diabetic retinopathy. In this study, we used streptozotocin (STZ)-induced diabetes in the rat to evaluate the expression of Slit2 and its receptor, Robo1, in background diabetic retinopathy. Recombinant N-Slit2 protein, the functional fragment of Slit2 protein, was used to study the effects of Slit2 on the expression of VEGF in rat’ retina. Additionally, FVMs and vitreous fluid from patients both with proliferative diabetic retinopathy (PDR) and without (control) were used to investigate the expression of Slit2 and Robo1 in the proliferative stage of DR.

## Materials and methods

### Animals and experimental diabetes

This study was approved by Peking University People’s Hospital Ethics Committee, complied with the Declaration of Helsinki. Animals were purchased from the Laboratory Animal Center, Peking University People’s Hospital. Animal care and experiments were conducted under institutional guidelines and food and tap water were given ad libitum. A total of eighty male Sprague Dawley (SD) rats (blood glucose concentrations 4–8 mM) were used. All protocols involving the use of rat adhered to the regulations set forth in the ARVO Statement for the Use of Animals in Ophthalmic and Vision Research. Forty-eight healthy SD rats (blood glucose concentrations 4–8 mM) of 8 weeks of age and weighing 200–250 g were used for the diabetic model. Diabetes was induced by a single intraperitoneal injection of STZ (Sigma-Aldrich, Co. St. Louis, MO, 70 mg/kg body weight, dissolved in citric buffer, pH 4.5) and confirmed by a measurement of blood glucose exceeding 25 mM for three consecutive days. The body weight and blood glucose were detected every two weeks. If the blood glucose were found to become normal concentrations, this rat was removed. Eight rats were excluded from the experiment because they failed to develop diabetes. We chose twelve rats at each time point in diabetic rats and ten rats at each time point in age- matched normal rats. The rats were sacrificed under anesthesia 1, 3 or 6 months after the onset of diabetes and the retinas were harvested. All rats were deeply anesthetized by an intraperitoneal injection of pentobarbital sodium to alleviate suffering. And the age-matched rats were used for the control group at 1, 3, 6 months.

### Intravitreal injections

Intravitreous injections were performed with a 30-gauge needle on a microsyringe (Hamilton, Reno, NV), by inserting the needle into the vitreous of rats at a site 1mm posterior to the limbus of the eye. Insertion and infusion were performed and directly viewed through an operating microscope. Care was taken not to injure the lens or the retina. The tip of the needle was positioned over the optic disk. Recombinant N-Slit2 protein, 50 ng per eye, was dissolved in an equal volume of 5-ul. N-Slit2 protein was injected to diabetic rats, Sham injections (phosphate buffered saline) were performed to both nondiabetic control rats as well as the untreated diabetic rats. The 5-ul volume was slowly injected into the vitreous. Any eyes that exhibited damage to the lens or retina were discarded and not used for the analyses[19]. Rats were killed on the day of 4 weeks, and the eyes were removed.

### Retinal VEGF measurement by enzyme-linked immunosorbent assay (ELISA)

Each retina was homogenized in 100 μl of solution consisting of 20mM imidazole hydrochloride, 100mM KCl, 1mM MgCl, 1mM EGTA, 1% Triton, 10mM NaF, 1mM sodium molybdinate, and 1mM EDTA. The solution was supplemented with a cocktail of protease inhibitors (Roche, Basel, Switzerland) before use. Samples were cleared via centrifugation for 10 min at 13,000 rpm. VEGF levels in retinal supernatants were determined using the ELISA kits according to the manufacturer’s instructions (R&D Systems, Minneapolis, MN)

### Vitrectomy, specimen collection and Slit2 measurement by ELISA

The study protocol was approved by the Ethics Committee of the Peking University People’s Hospital, and informed consent was obtained from all patients according to the World Medical Association Declaration of Helsinki. This prospective, comparative study included patients with proliferative diabetic retinopathy caused by diabetes mellitus type 2 (study group) and those with idiopathic macular holes or preretinal membranes (control group).All the samples were collected from March 2010 to March 2013.Vitreous samples were collected following Tao’s methods[20].We measured these specimens from 2013 to 2014. The researcher can not identify the individual participants during or after data collection. The concentrations of Slit2 were measured using an enzyme-linked immunosorbent assay (human Slit2 ELISA Kit; Groundwork Biotechnology Diagnosticate, San Diego, CA). The measurements were conducted according to the manufacturer's instructions. The FVM specimens were surgically removed from the eyes of 11 patients with type 2 diabetes with PDR (11 eyes). Six FVM specimens that were obtained and immediately put into liquid nitrogen were processed for RT–PCR analysis. The remaining five FVM specimens were fixed in 4% paraformaldehyde (PFA) and subsequently embedded in optimum cutting temperature compound in preparation for immunohistochemistry. In a similar manner, fibrous membranes were obtained from ten eyes with preretinal membranes, and these served as controls.

### Immunohistochemistry

Paraffin-embedded rat eyeball tissues were cut at 6 um thickness and de-paraffinized with xylene and a graded series of ethanol solutions. Endogenous peroxidase activity was eliminated by incubating the retinas in 3% hydrogen peroxidase in PBS for 20 min at room temperature. After being washed with PBS, the slides were incubated with 1% bovine serum albumin (BSA) for 1 h to block non-specific labeling, followed respectively in incubation with antibodies against both Slit2 (Millipore, Temecula, CA) and Robo1 (Abcam, Cambridge, UK) at 1:300 dilutions in 0.3% BSA overnight at 4°C. Following incubation with the primary antibody, the slides were washed with PBS three times and incubated for 20 min with biotin- conjugated secondary antibody (Envision-Detection Kit, GK500705, Gene Tech) at room temperature. The slides were then washed three times in PBS, and the antibody complexes were viewed using diaminobenzidine (DAB). Images of slides were captured on an upright Nikon microscope (Eclipse E800) using Spot advanced software (Spot Diagnose Instruments V4.0.1). In each case, preimmune IgG and secondary control incubations were conducted to determine specificity of staining.

### Dual-color immunofluorescence staining

Human fibrovascular membrane tissues were snap-frozen and 6 μm sections were cut. Thawed tissue sections were air dried, placed in 4% PFA for 20 min for fixing, washed with PBS, and blocked with 10% normal goat serum for 1 h at 37°C. Next, 1:100 anti- VEGF antibody (Santa Cruz, Santa Cruz, CA) in combination with either 1:200 anti-Slit2 polyclonal antibody (Millipore, Temecula, CA) or 1:100 anti-Robo1 polyclonal antibody (Abcam, Cambridge, UK) together at the same time were applied to the tissue sections at 4°C overnight and incubated for 1 h at 37°C with 1:100 fluorescein isothiocyanate (FITC)-conjugated goat anti-rabbit and tetramethyl rhodamine isothiocyanate (TRITC)-conjugated goat anti-mouse secondary antibodies (Santa Cruz, CA, USA) as appropriate. Following incubation, the slides were washed, and cell nuclei were stained with 4’, 6’-diamino-2- phenylindole (DAPI). Images were acquired using a fluorescence microscope equipped with a digital camera. For each of the immunostaining procedures, negative controls included the omission of the primary antibody and the use of an irrelevant polyclonal or isotype-matched monoclonal primary antibody. In all cases, negative controls showed only faint, insignificant staining.

### Western blot analysis

Protein extracts from retinal tissues were prepared at different time points on ice using a protein extraction kit and protease inhibitor kit (Pierce, Rockford,IL). Protein extracts were sonicated in 500ul lysis buffer. The homogenates were centrifuged at 12,000 g for 20 min at 4°C. The supernatants were collected, transferred to a fresh tube and stored at -80°C in preparation for SDS-PAGE(sodium dodecyl sulfate-Polyacrylamide gel electrophoresis). The protein content of each lysate was measured using a BCA Protein Assay Kit (Tianlaishengwu, tianlai, China) according to the manufacturer’s directions with BSA as the standard (Bio-Rad Laboratories, Hercules, CA). The samples were then boiled for 5 min in sample buffer. Equal amounts of protein were loaded and analyzed by immunoblotting. The primary antibodies anit-Slit2 (1:1000; Millipore, Temecula, CA), anti-Robo1 (1:500; Abcam, Cambridge, UK) and anti-β-actin (1:1000; Boster, China) were used. Membranes were washed and incubated with peroxidase-conjugated secondary antibodies (1:6000; Boster, China), Western blot image was analyzed by a tool called “Image J”. The band densities of the Slit2 and Robo1 proteins were normalized to each β-actin internal control. Western blots were repeated three times, and qualitatively similar results were obtained.

### Reverse-transcription PCR

Total RNA was isolated from the whole rat retinas or human fibrovascular membrane tissues with Trizol reagent (Invitrogen, Carlsbad, CA) according to the manufacturer’s instructions. Two micrograms of RNA were converted into cDNA. The mixture was incubated for 60 min at 42°C and reverse transcription was terminated by incubation at 95°C for 5 min. The single-stranded cDNA was amplified by PCR utilizing sequence-specific primers for human β-actin (144 bp): sense: CTTAGTTGCGTTACACCCTT, antisense: CCTTCACCGT TCCAGTTT; human Slit2 (107bp): sense: CACCTCGTACAGCC GCACTT, antisense: TGTGGACCGCTGAGGAGCAA; human Robo1 (104bp): sense: GGAAGAAGACGAAGCCGACAT, antisense: TCTCCAGGTCCC CAACACTG; and human VEGF (110bp): sense: AGTTCCACCACCAAACATGC, antisense: TGAAGGGACACAAC GACACA. 25cycles (β-actin and Slit2) or 30 cycles (VEGF and Robo1) were used.

### Real-time PCR

The real-time PCR assays were performed using IQ Supermix (Bio-Rad, Hercules, CA) with each 20 ul reaction mixture containing 2 ul cDNA, 7.2 ul sterilized water, 10 ul SYBR Green real-time PCR Master Mix (2X), and 0.8 ul of each primer (10 Mm), utilizing sequence-specific primers for rat β-actin (222bp): sense: AGCCATG TACGTAGCCATCC, antisense: GCTGTGGTGGTGAAGCTGTA; rat Slit2 (187 bp): sense: GTGTGCTTCTGTCTGCTCTC, antisense: GCCAATGCCACTCATCTTCC; and rat Robo1 (195 bp): sense: CACCGAATGCTGCTGCCAAGT, antisense: CGCCACCATAACATCTGAAGG. Amplification was carried out in 96-well plates on an iCycler iQ real-time detection system (Bio-Rad). Thermo-cycling conditions consisted of 3 min at 95°C for activating the iTaq DNA polymerase and 35 cycles of a 20 sec, 95°C denaturation step; a 15 sec, 61°C annealing step; and a 15 sec, 72°C extension step. Each PCR experiment included one synthetic template control and one no-template control for each target. The specificity of the amplification reactions was first confirmed by agarose gel electrophoresis and subsequently by melting curve analysis. The expression of Robo1 and Slit2 was normalized to β-actin expression and calculated using the equation: Fold change = 2^−ΔΔct^.

### Statistical analysis

The expression of Slit2 and Robo1 in rat retina are presented as the mean ± SD calculated from triplicate experiments, as described in the figure legends. Statistical differences were evaluated using the one-way ANOVA followed by a least significant difference (LSD) post hoc test using SPSS 10.0 (SPSS Inc., Chicago, IL). Statistical significance was defined as a *P* value <0.05. Measurement of the Slit2 concentration was evaluated using the Mann-Whitney rank-sum test. Two-tailed *P*<0.05 was considered to indicate statistical significance.

## Results

### 1. Quantification of Slit2 and Robo1 mRNA expression in the rat retina

**Fig 1:**
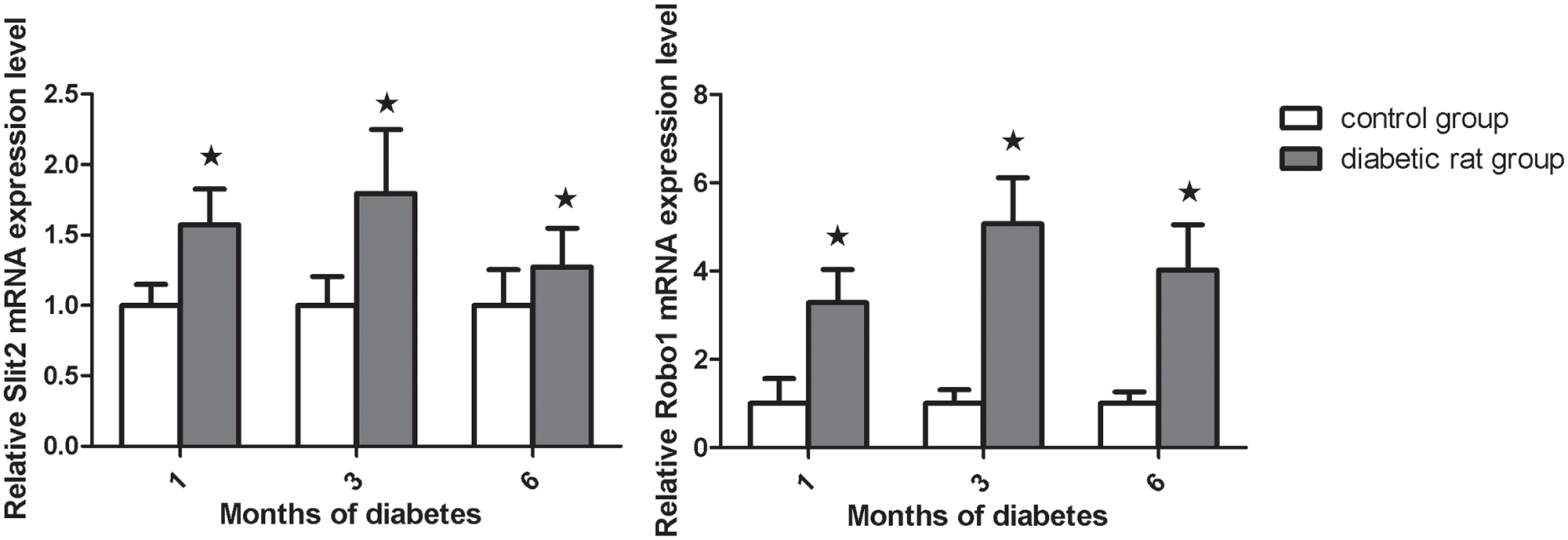
The expression of Slit2 and Robo1 mRNA in the STZ diabetic rat retinas relative to the control rat retinas as measured by real-time PCR. A: The expression of Slit2 mRNA was increased after 1, 3 and 6 months of diabetes than age matched control group. (*P*<0.05). B: The expression of Robo1 mRNA was significantly increased after 1, 3 and 6 months compared to controls. Values are the means ± SD of at least three independent experiments. Asterisks denote values significantly different between the diabetic and control groups (P<0.05). The relative expression level of the control group cell was set to 1.

The expected 187 bp and 195 bp PCR products, representative of Slit2 and Robo1, respectively, were observed, demonstrating that Slit2 and Robo1 were expressed in the rat retinal tissue. The Slit2 and Robo1 expression profiles normalized for β-actin were fairly uniform in the retinal specimens. (Fig 1) The expression of Slit2 mRNA increased in the first month (1.57 fold, *P*<0.05). Real -time RT-PCR demonstrated that Slit2 mRNA expression peaked at 3 months (1.79 fold, *P*<0.01); expression was still found to increased at 6 months (1.27 fold, *P*<0.01) (Fig 1A). The expression of Robo1 mRNA was similar to that of Slit2. Robo1 expression in the diabetic rats was significantly higher than in the controls at 1 (3.28 fold, *P*<0.05), 3 months (5.07fold, *P*<0.05), and 6 months (4.02fold, P<0.01) (Fig 1B).

### 2. Western blot analysis of Slit2 and Robo1 expression in the rat retina

**Fig 2:**
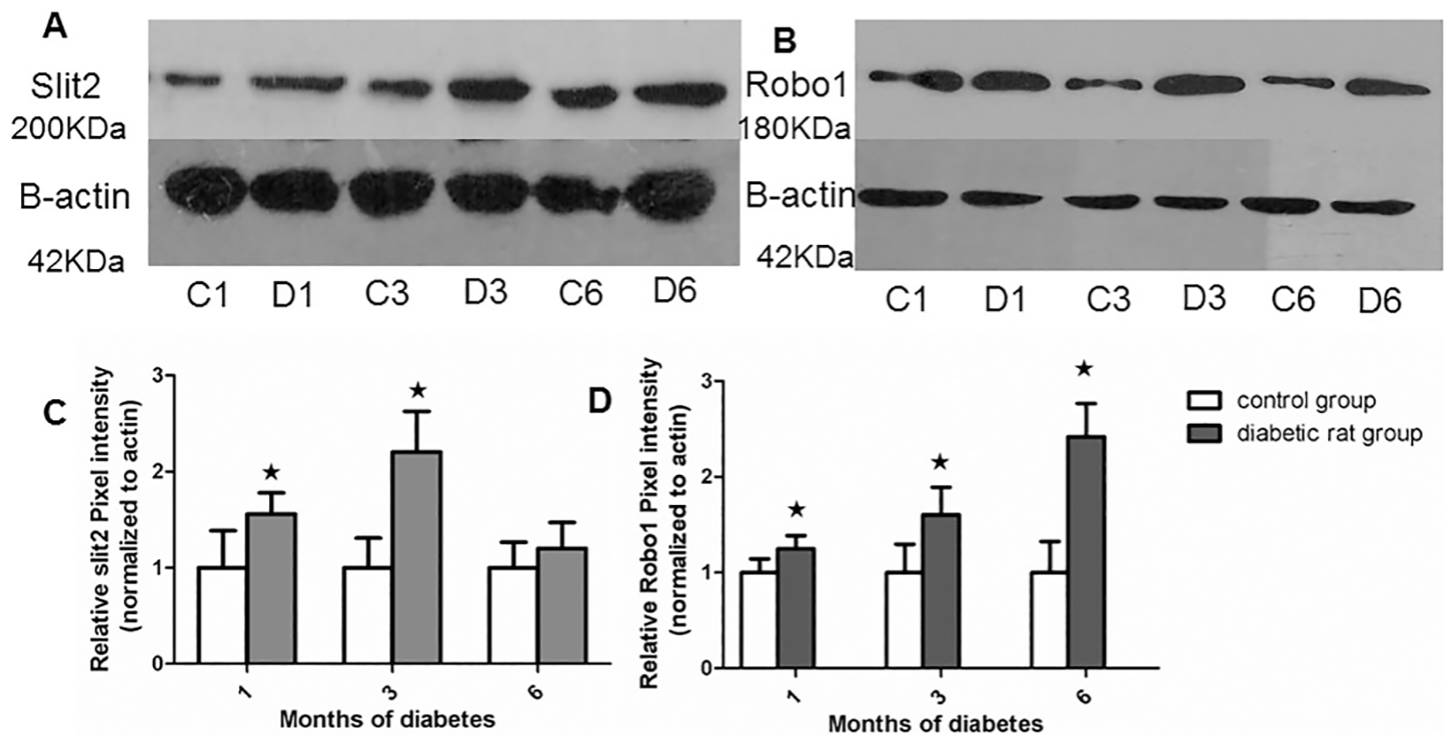
The expression of Slit2 and Robo1 protein in the retinas of STZ diabetic rats relative to the control rat retinas as measured by immunoblotting and normalized to β-actin expression. A: A representative photograph of the western-blot analysis of Slit2 expression. B: A representative photograph of the western-blot analysis of Robo1 expression. C: The relative Slit2 protein levels in the age matched control group and diabetic rats after 1, 3 and 6 months. D: The relative Robo1 protein levels in the age matched control group and diabetic rats after 1, 3 and 6 months. Values are the means ±SD of at least three independent experiments. Asterisks denote values significantly different from the diabetic rat group compared to the control group (p<0.05). Slit2 expression after 1 and 3 months was significantly increased compared to controls (p<0.05); however, slit2 did not vary significantly at 6months compared to controls (p>0.05). Robo1 expression after 1, 3 and 6 months was significantly increased compared to controls (p<0.05); Western blot image was analyzed by a tool called “Image J”. The pixel intensity of the control group was set to 100%. C1, C3, C6: control group1, 3, 6 months; D1, D3, D6:diabetic rat group1, 3, 6 months.

Western blot analysis of whole retina protein samples resulted in the immunodetection of two bands that migrated at approximately 200 KDa (Slit2) (Fig 2A) and180 KDa (Robo1) (Fig 2B). The levels of both Slit2 and Robo1 varied in a time-dependent manner. We found that Slit2 began to increase after 1 month (1.56 fold, *P* < 0.05) and was more elevated after 3 months (2.20 fold, *P*<0.05). After 6 months, Slit2 expression was increased compared to its control group, but this change did not have significance (*P*> 0.05) (Fig 2C). The expression of Robo1 protein was similarly altered in a time-dependent manner. Robo1 protein expression increased after 1 month (1.24 fold, *P* < 0.05) and peaked after 3 months (1.60 fold, *P*<0.05). It was observed to increase after 6 months (*P*<0.05) (Fig 2D).

### 3. Localization of Slit2 and Robo1 immunoreactivity in the rat retina

**Fig 3:**
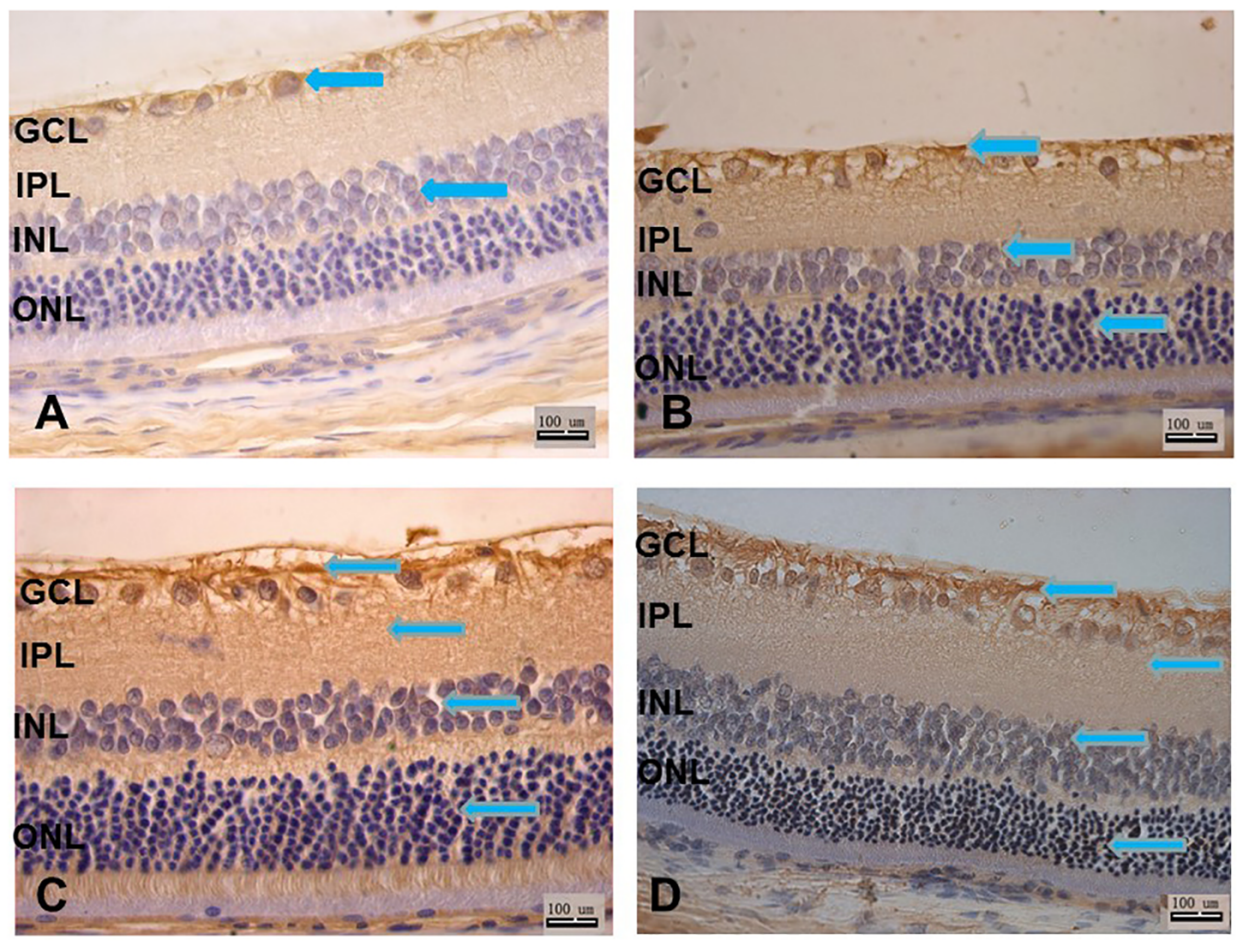
A–D: Slit2 antibody staining of rat retina sections. A: control group; B: 1 month after diabetes induction; C: 3 months after diabetes induction; D: 6 months after diabetes induction. Scale bar 100 μm. GCL, ganglion cell layer; IPL, inner plexiform layer; INL, inner nuclear layer; ONL, outer nuclear layer.

**Fig 4:**
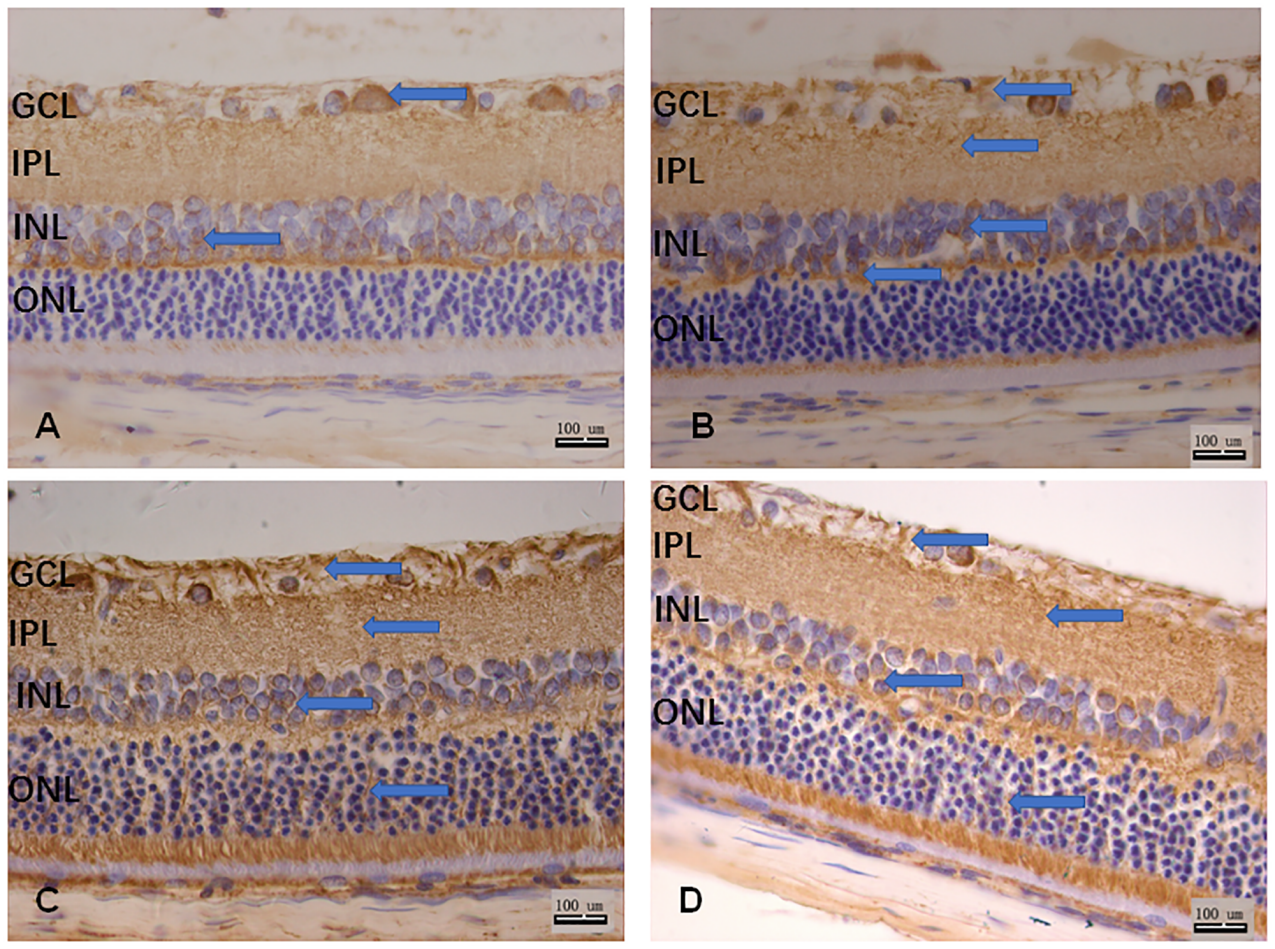
A–D: Robo1 antibody staining of rat retina sections. A: control group; B: 1 month after diabetes induction; C: 3 months after diabetes induction; D: 6 months after diabetes induction. Scale bar 100 μm. GCL, ganglion cell layer; IPL, inner plexiform layer; INL, inner nuclear layer; ONL, outer nuclear layer.

In control SD rats, Slit2 was observed in the ganglion cell layer, the nerve fiber layer and the inner nuclear layer (Fig 3A). Robo1 expression was localized to the ganglion cell layer, the nerve fiber layer, and the putative inner nuclear layer (Fig 4A). Both the expression of Slit2 and Robo1 were only weak and insignificant.

In the first month after diabetes induction, Slit2 was expressed in the ganglion cell layer, nerve fiber layer, the inner nuclear layer and the outer nuclear layer (Fig 3B), while Robo1 positive staining was observed in the ganglion cell layer, the nerve fiber layer, the inner nuclear layer, the inner plexiform layer and the inner segment of the photoreceptor layer (Fig 4B). The expression of Slit2 and Robo1 was stronger 1 month after induction than it was in the control rats.

The localization of Slit2 was observed in the ganglion cell layer, the nerve fiber layer, the inner nuclear layer, the outer nuclear layer and the inner segment of the photoreceptor layer (Fig 3C). The Robo1 expression pattern after 3 months was similar to that after 1 month, with positive staining in the ganglion cell layer, the nerve fiber layer, the inner nuclear layer, the inner plexiform layer and the inner segment of the photoreceptor layer. However, at this time the outer nuclear layer began to stain positively (Fig 4C).

At 6 months after diabetes induction, both the Slit2 (Fig 3D) and the Robo1 (Fig 4D) expression patterns remained the same as at 3 months, with the exception that expression in the outer nuclear layer was decreased.

### 4. The concentrations of VEGF in the rat’s retina

**Fig 5:**
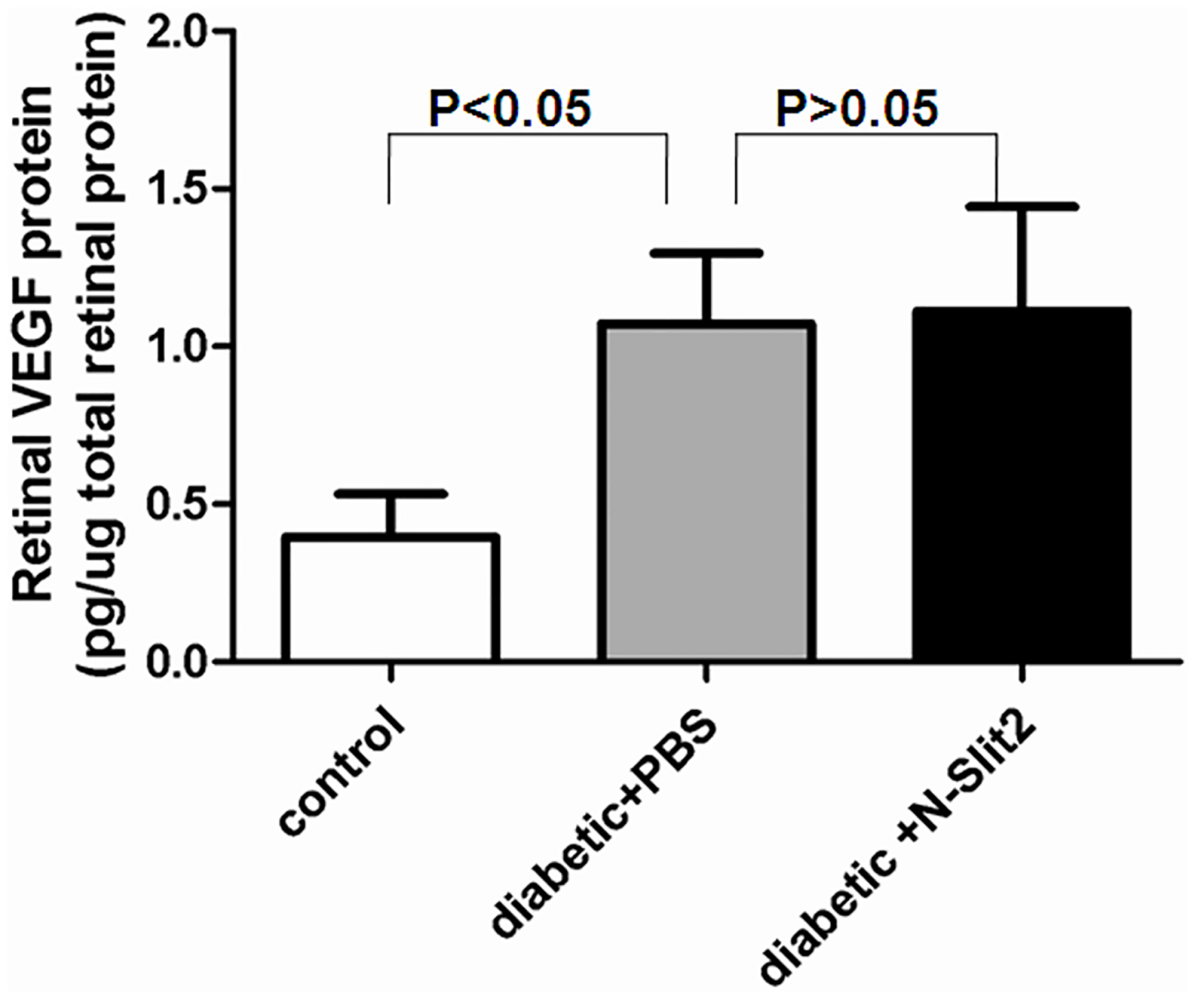
The retinal levels of VEGF were quantified by using the ELISA technique. Compared with the retina of control group, the retina of diabetic animals demonstrate a 2.71-fold increase in VEGF levels (P<0.05). Diabetic animals of treatment with recombinant N-Slit2 protein does not significantly increase the retinal VEGF levels (P>0.05).

VEGF is a major potent angiogenic factor in the diabetic retina. We measured VEGF retinal protein levels via ELISA. Compared with the retina of non-diabetic animals, the retina of diabetic animals demonstrated a 2.71-fold increase in VEGF levels (0.39 ± 0.09 pg/ug vs. 1.07 ± 0.22 pg/ug of retinal protein; P<0.001, n = 6) (Fig 5). The VEGF levels in diabetic animals treated with recombinant N-Slit2 protein did not increase significantly compared to the levels in the untreated diabetic animals. (P>0.05, n = 6)

### 5. Characteristics of the patients

The study included 55 patients, with 27 patients in the DR group (23 patients with vitreous hemorrhage and 4 patients without vitreous hemorrhage)and 28 patients in the control group (Table 1). There were no statistically significant differences in age, gender, prevalence of arterial hypertension. The DR group has their independent features, including the duration of diabetes, fasting blood glucose, glycosylated hemoglobin, in the ratio of previous panretinal photocoagulation, anterior chamber neovascularization, vitreous hemorrhage, fibroneovascular membranes, or tractional retinal detachment. In the study group, panretinal photocoagulation had been performed in 8 (30%) patients at least 4 months prior to inclusion in the study.

**Table 1:** Composition of the study population.

	Proliferative Diabetic Retinopathy(*n* = 27)	Non-diabetic Ocular Diseases (*n* = 28)	P
**Age, y**	**51.59 ± 14.22**	**52.29±12.63**	**0.849**
**Female sex, *n* (%)**	**12 (44)**	**13 (46)**	**0.883**
**Hypertension, *n* (%)**	**17 (63)**	**12 (43)**	**0.135**
**Duration of diabetes, y**	**10.3 ±8.27**	**—**	**—**
**Fasting blood glucose, mmol/L**	**7.25±1.98**	**—**	**—**
**Glycosylated hemoglobin**	**7.95 ± 1.75**	**—**	**—**
**Subgroups, *n*, %**			
**Panretinal photocoagulation history**	**8 (30)**	**—**	**—**
**Anterior chamber neovascularization**	**3 (11)**	**—**	**—**
**Vitreous hemorrhage**	**23 (85)**	**—**	**—**
**Fibroneovascular membranes**	**24 (89)**	**—**	**—**
**Tractional retinal detachment**	**20 (74)**	**—**	**—**
**Idiopathic macular hole**	**—**	**17 (61)**	**—**
**Idiopathic epiretinal membrane**	**—**	**11 (39)**	**—**

Values are mean ±SD except where indicated.

### 6. The concentrations of Slit2 in the patient’s vitreous

**Fig 6:**
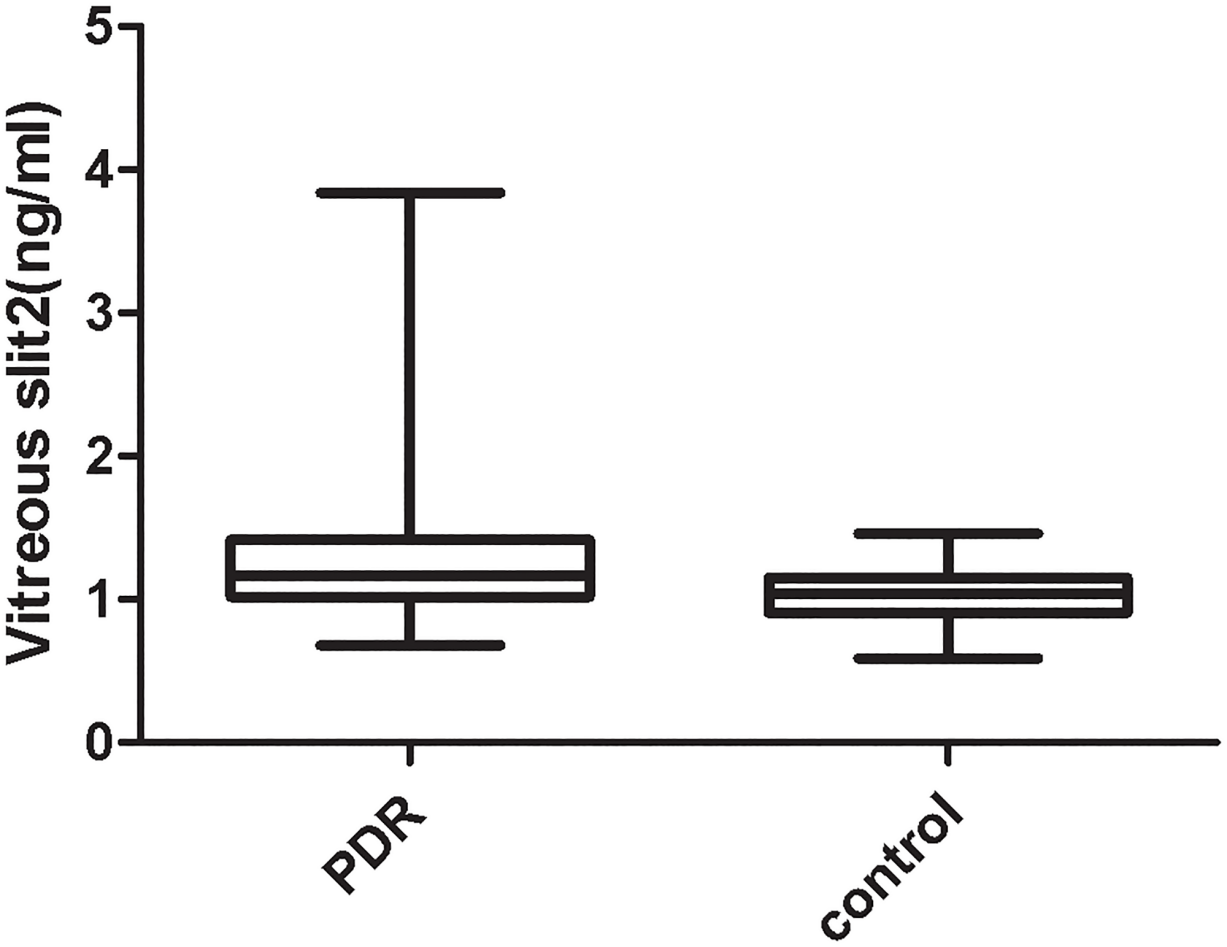
The vitreous concentration of Slit2 in patients with PDR and in patients with idiopathic preretinal membrane or macular holes. Box plots showing the vitreous concentration of Slit2 in patients with PDR and in patients with idiopathic preretinal membrane or macular holes, with a statistically significant difference between the groups (P<0.05).

**Fig 7:**
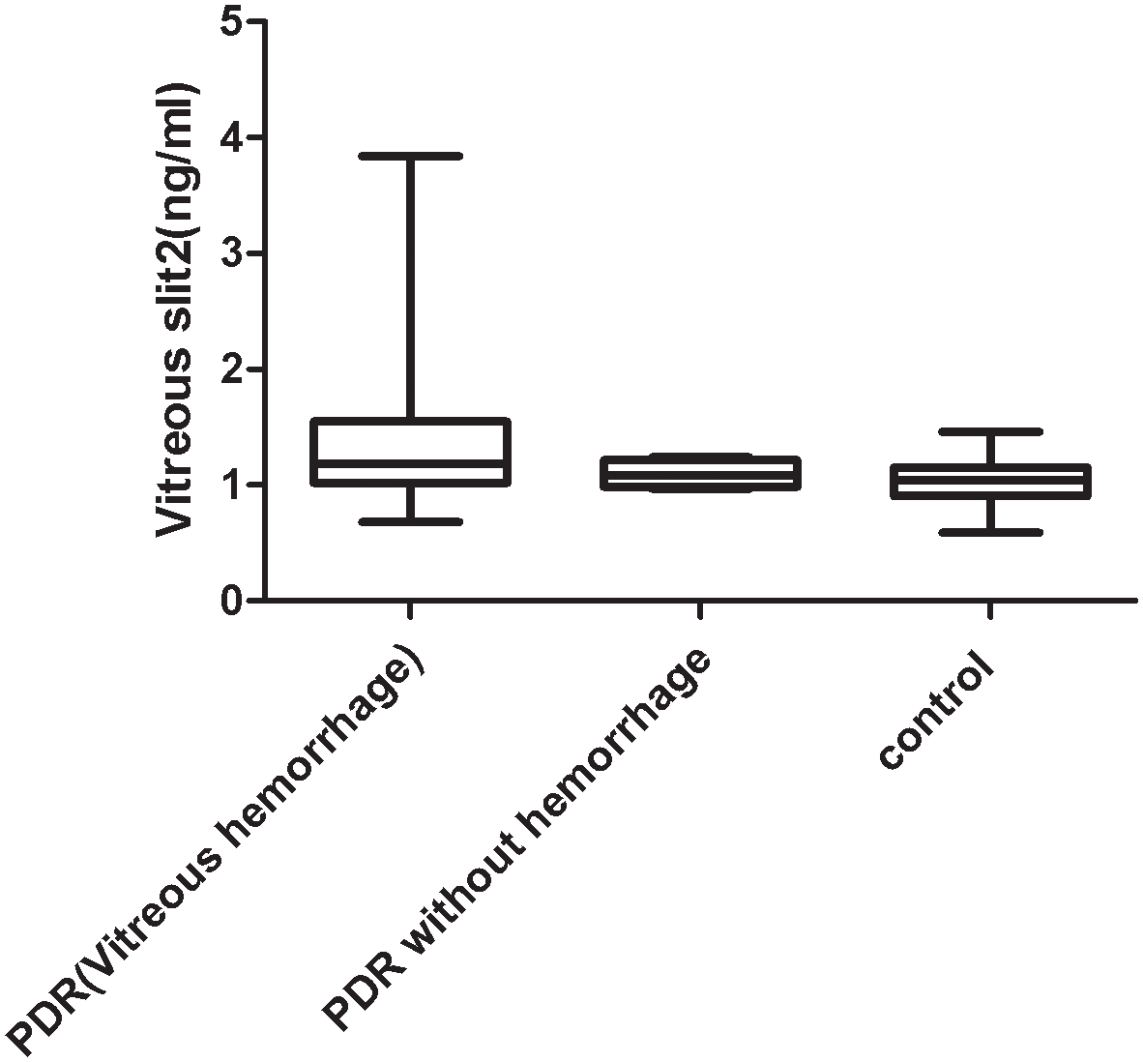
The vitreous concentration of Slit2 in different patients. Box plots showing the vitreous concentration of Slit2 in patients with PDR (23 patients with vitreous hemorrhage and 4 patients without vitreous hemorrhage) and in 28 patients with idiopathic preretinal membrane or macular holes, with a statistically significant difference between PDR with vitreous hemorrhage and patients with idiopathic preretinal membrane or macular holes, (*P*<0.05),However, there is no difference between proliferative diabetic retinopathy with vitreous hemorrhage or not.(*P*>0.05)

Fig 6 shows the concentrations of vitreous Slit2 in the PDR and control groups. Concentrations of vitreous Slit2 ranged from 0.68–3.84 ng/mL in patients with proliferative vitreoretinopathy and 0.59–1.46 ng/mL in non-diabetic control subjects. Vitreous concentrations of Slit2 were significantly higher in the DR group than in the control group (1.34±0.62 ng/mL vs. 1.05±0.19 ng/mL; *P* = 0.015). Fig 7 shows the concentrations of vitreous Slit2 in the PDR with or without vitreous hemorrhage. Concentrations of vitreous Slit2 ranged from 0.68–3.84 ng/mL in patients with vitreous hemorrhage and 0.97–1.24 ng/mL in subjects without vitreous hemorrhage. Vitreous concentrations of Slit2 were significantly higher in the DR with vitreous hemorrhage group than in the control group (*P*<0.05), However, the concentrations of Slit2 have no difference between DR patient with or without vitreous hemorrhage.

### 7. Immunohistochemical detection of Slit2 and Robo1 in human FVMs and preretinal membranes

**Fig 8:**
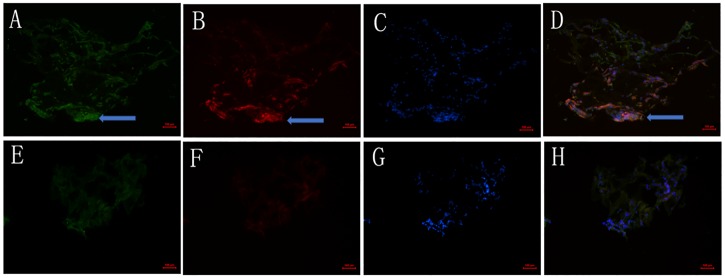
Expression of Slit2 and VEGF in the FVMs of eyes with PDR and the preretinal membranes of control patients without DR. (A-D) Immunostaining for Slit2 (A), VEGF (B), and DAPI (C) in FVMs from eyes with PDR. Staining intensities of Slit2 were strong (A) and co-localized with VEGF (D). (E-H) Staining of Slit2 (E), VEGF (F), and DAPI (G) in the preretinal membranes of control patients without DR. Weak staining of Slit2 (E) and insignificant staining of VEGF (F) were observed. Scale bar 50 μm.

**Fig 9:**
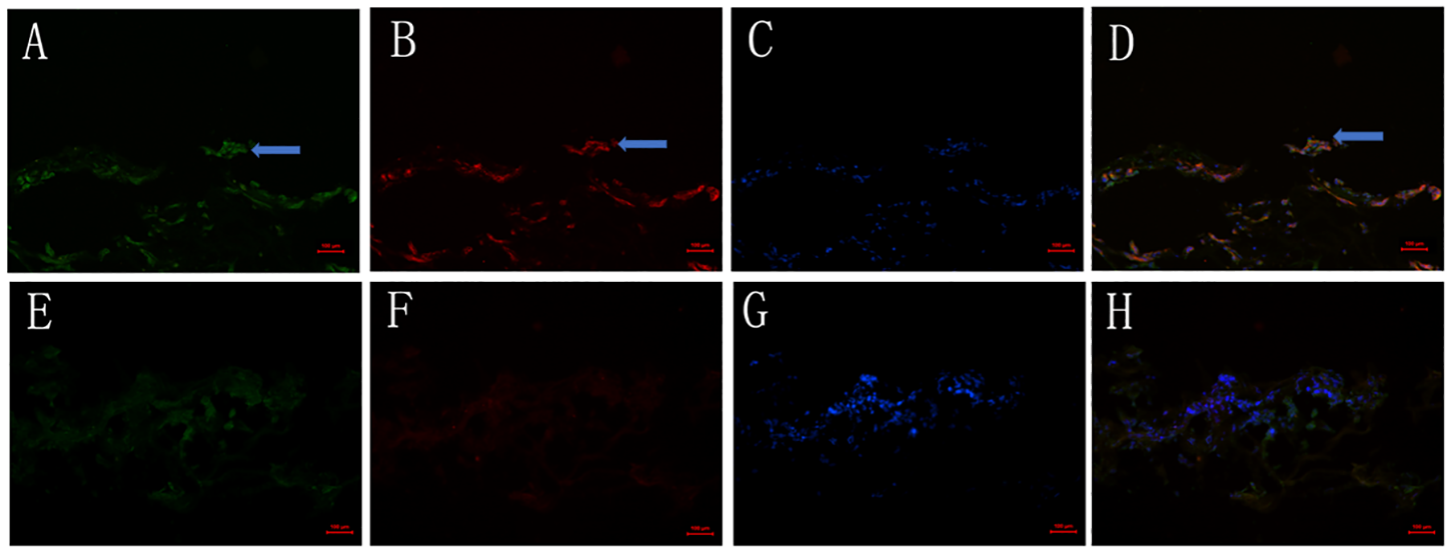
Expression of Robo1 and VEGF in the FVMs of eyes with PDR and the preretinal membranes of control patients without DR. (A-D) Immunostaining for Robo1 (A), VEGF (B), and DAPI (C) inFVMs from eyes with PDR. Staining intensities of Robo1 were strong (A) and co-localized with VEGF (D). (E-H) Staining of Robo1 (E), VEGF (F), and DAPI (G) in the preretinal membranes of control patients without DR. Weak staining of Robo1 (E) and insignificant staining of VEGF (F) were observed. Scale bar 50 μm.

The expression of Slit2, Robo1 and VEGF was detected in all the tissues of the FVMs with PDR. The majority of Slit2 (Fig 8A) and VEGF (Fig 8B) or Robo1 (Fig 9A) and VEGF (Fig 9B) signals co-localized in all specimens of the DR group. The membranes removed from the eyes of the control group showed insignificant staining of VEGF (Figs 8F and 9F). The expression of Slit2 (Fig 8E) and Robo1 (Fig 9E) in the control group was weak or insignificant. The nuclei were stained with DAPI (Figs 8C, 8G, 9C and 9G).

### 8. The expression of Slit2, Robo1 and VEGF mRNA in the human FVMs and preretinal membranes

**Fig 10:**
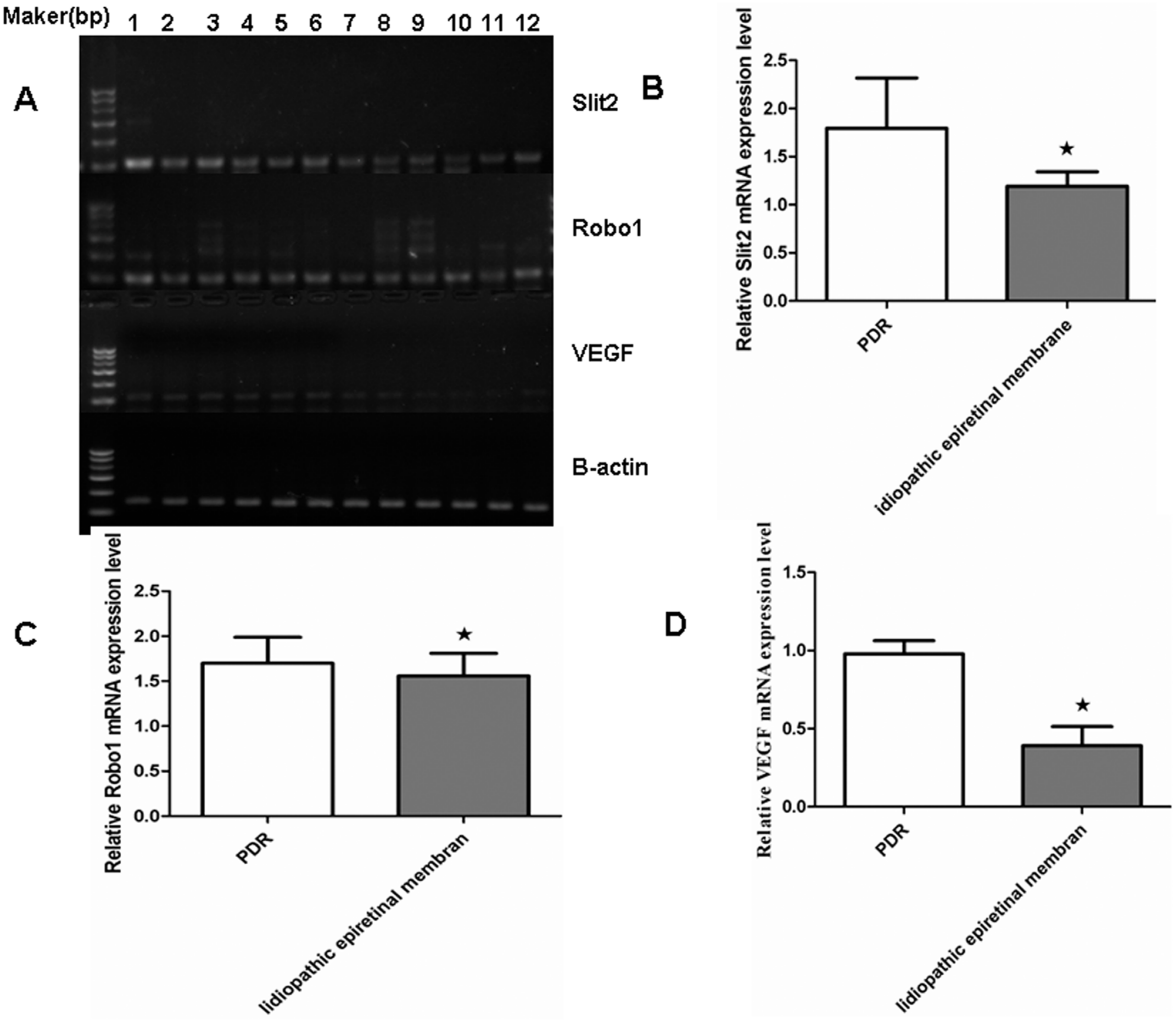
RT-PCR analysis showed the mRNA expression of Slit2, Robo1, VEGF, and ß-actin in the FVMs of different patients. A: RT-PCR amplification of Slit2, Robo1, VEGF, and ß-actin in the FVMs of six patients with PDR (panels 1–6) and in the membranes of six patients with idiopathic epiretinal membranes (panels 7–12).B: A representative photograph of the mRNA analysis of Slit2 expression. C: A representative photograph of the mRNA analysis analysis of Robo1 expression. D: A representative photograph of the mRNA analysis analysis of VEGF expression. Asterisks denote values significantly different to the control group (*P*<0.05).

The results of the semi-quantitative RT-PCR analysis demonstrate that the expressions of Slit2 mRNA (Fig 10B, *P* <0.05), Robo1 mRNA (Fig 10C, *P*< 0.05), and VEGF mRNA (Fig 10D, *P* < 0.05) were significantly higher in the FVMs of the six patients with PDR than in the membranes of the six patients with idiopathic epiretinal membranes (Fig 10).

## Discussion

Slit-Robo signal was first discovered as a major repellent pathway of the central nervous systems [21]. Recently, it was found to play an important role in angiogenesis, including ocular angiogenesis. In our previous studies, we reported the first observation of Robo1 and Robo4 mRNA and protein expressed in the FVMs of human eyes with PDR. Additionally, these genes can be expressed in RF/6A and RPE cells[22–26]. The findings of this study indicate that STZ-induced diabetes in rats resulted in the time-dependent alteration of the expression of both Slit2 and Robo1 in retinal tissue. Recombinant N-Slit2 protein, the functional fragment of Slit2 protein, did not increase the expression of VEGF in diabetic rat’s retina. In our study, the co-localization of VEGF with Slit2 or Robo1 were observed in the fibrovascular membranes of the eyes with PDR. Slit2 and Robo1 mRNA levels were significantly higher in the FVMs of eyes with PDR than in the eyes of the control group. Moreover, the vitreous concentrations of Slit2 were significantly higher in eyes with PDR than in non-diabetic eyes. These data suggest that the Slit-Robo signal may be involved in retinal neovascularization independent of VEGF during the development of DR.

DR is the most common microvascular complication for patients with chronic diabetes[27]. The STZ-induced diabetic rat displays similar retinal changes to those observed in the early stage of human DR[28]. Many studies have reported that rats living with induced diabetes for prolonged periods, for example 3–8 months, have vascular pathologies such as vascular permeability[29], the appearance of acellular capillaries[30], and endothelial apoptosis[31]. In the present study, we examined rats 1, 3, or 6 months after the induction of diabetes. Using this diabetic rat model, we report the first examination of the role of the Slit-Robo signal in diabetic retina in the early stages of induced diabetes.

Slit2 and Robo1 were thought to be the most important factors in the Slit-Robo family[32]. Recombinant Slit2 protein was documented to attract endothelial cells and promote tube formation in a Robo1-dependent manner[33]. Previous studies of Robo1 have primarily concentrated on organ development and tumor tissue, but recently there have been reports of robo1 expression in human eye tissue[24, 25]. Previously, we determined that Robo1 performs a role in the angiogenic processes associated with the normal development of murine retinal vasculature[34]. Here, the Slit-Robo signal was studied at both mRNA and protein levels. We found that the levels of both Slit2 and Robo1 mRNA and proteins were altered in a time-dependent manner in the STZ- induced diabetic rats. Both were increased at 1, 3 and 6 months comparing to the age- matched control group. The Slit2 localization was simultaneously observed in the ganglion cell layer, the nerve fiber layer, the inner nuclear layer, the outer nuclear layer and the inner segment of the photoreceptor layer. We also observed Robo1-positive staining in the ganglion cell layer, the nerve fiber layer, the inner nuclear layer, the inner plexiform layer and the inner segment of the photoreceptor layer of diabetic rats. The alterations in the expression of Slit2 and Robo1 in the retina of STZ-induced diabetic rats have prompted us to explore new approaches to the prevention of cellular injury in the early diabetic retina. Further studies will be required to investigate the possible role of the Slit-Robo signal in early diabetes.

Currently, VEGF is thought to be the most potent angiogenic factor in neovascularization[35]. However, disappointing results from a clinical trial using several inhibitors for VEGF and its receptors have indicated that other angiogenic factors may be playing an important role in angiogenesis[36]. The interaction of the Slit-Robo signal with VEGF signaling is one possible alternate mechanism. Recombinant N-Slit2 protein is the functional fragment of Slit2 protein, little is known about its role on the expression of VEGF. So we gave the recombinant N-Slit2 protein to rat by intravitreous injections. We compared the VEGF levels in retinal supernatants by ELISAs. We found that N-Slit2 protein did not increase the expression of VEGF, so we think that Slit-Robo plays in DR, but this role is not by VEGF signaling pathway.

PDR is characterized by fibrovascular proliferation. The imbalance of angiogenic and anti-angiogenic factors in the membranes and vitreous is the major mechanism of this angiogenesis[37]. Alternatively, Slit2 may have a synergistic effect on the angiogenic activities of VEGF signaling. Because rodents do not develop proliferative diabetic retinopathy, we selected the patients with proliferative diabetic retinopathy (PDR) to examine the roe of Slit2 and Robo1 in proliferative stage. Our findings that the vitreous of eyes with proliferative diabetic retinopathy have stronger Slit2 expression suggests that Slit2 is a novel player in angiogenesis. FVMs with PDR were found to sites of the co-localization of VEGF and Slit2 or VEGF and Robo1, while the control group showed insignificant staining for VEGF. Likewise, the expression of Slit2 and Robo1 in the control group was weak or insignificant. The expression of Slit2, Robo1 and VEGF mRNAs was significantly higher in the FVMs of eyes with PDR than those of the control group. Together, these findings indicate that the Slit-Robo signal may be involved in PDR. These findings may help us better understand the pathogenesis of diabetic retinopathy and provide novel therapeutic targets.

In conclusion, the Slit-Robo signal plays an important role in both background and PDR. The details of Slit-Robo signaling are not fully understood, nor is the mechanism by which these signaling events affect the pathogenesis of ocular angiogenesis. Therefore, further investigation is required to better understand the role of Slit-Robo in angiogenesis and to develop novel therapeutic approaches for ocular angiogenesis diseases, including age related macular degeneration, DR and macular edema.

## Supporting information

S1 Fig The changes of blood glucose level in rats. SD: the normal Sprague-Dawley rats; DM: diabetic Sprague Dawley rats.
(TIF)

S2 Fig The changes of body weight in rats. SD: the normal Sprague-Dawley rats; DM: diabetic Sprague Dawley rats.
(TIF)

S1 File The data of the manuscript. This file included the data which revealed in the manuscript.
(ZIP)
