# Supplementary figures and images for: The expression of the Slit-Robo signal in the retina of diabetic rats and the vitreous or fibrovascular retinal membranes of patients with proliferative diabetic retinopathy
Source: PLoS One. 2017 Oct 3;12(10):e0185795. doi: 10.1371/journal.pone.0185795 (PMC5626485; doi:10.1371/journal.pone.0185795)

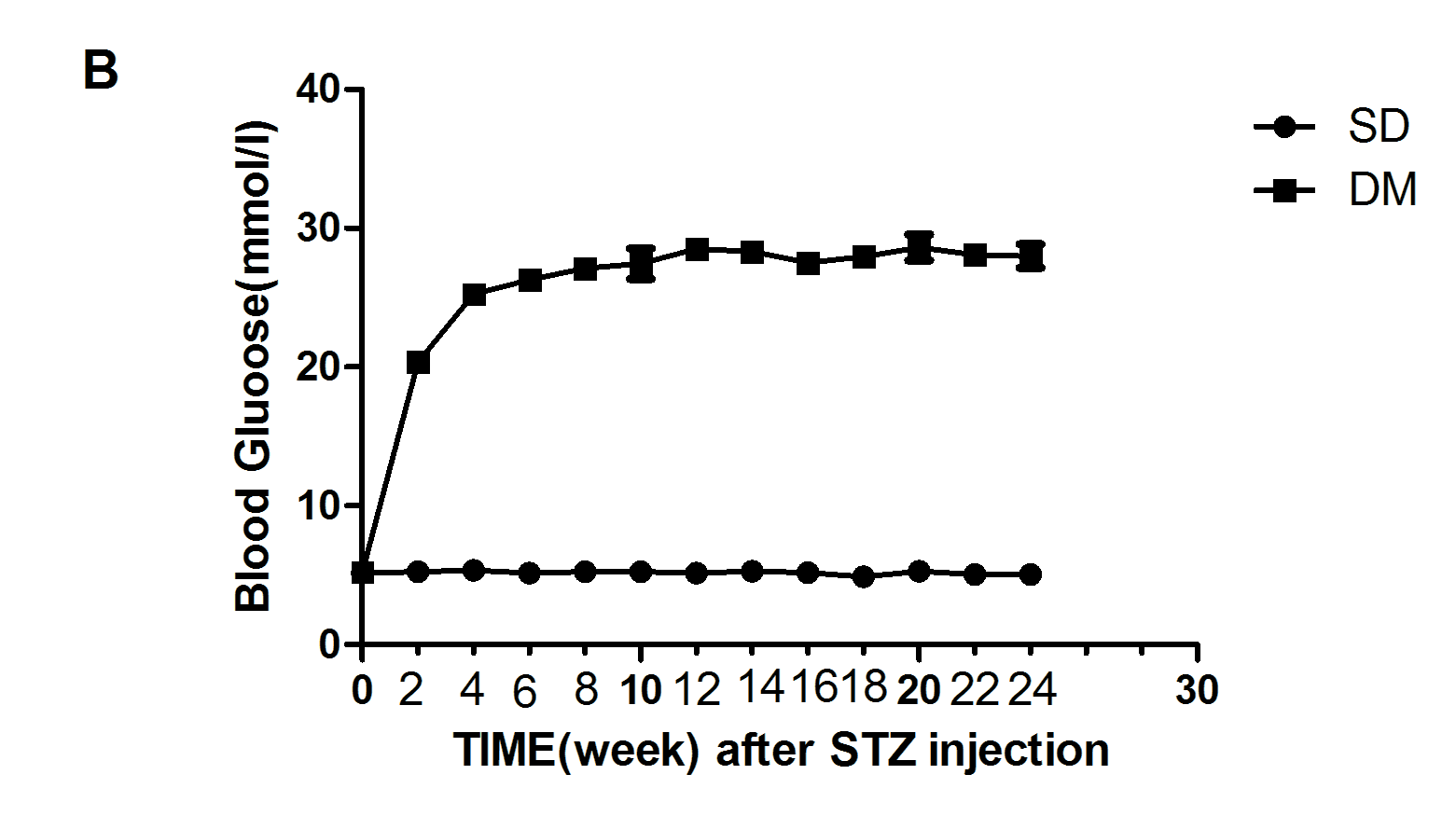

Supplement: S1 Fig — SD: the normal Sprague-Dawley rats; DM: diabetic Sprague Dawley rats. (TIF) [file pone.0185795.s001.tif]

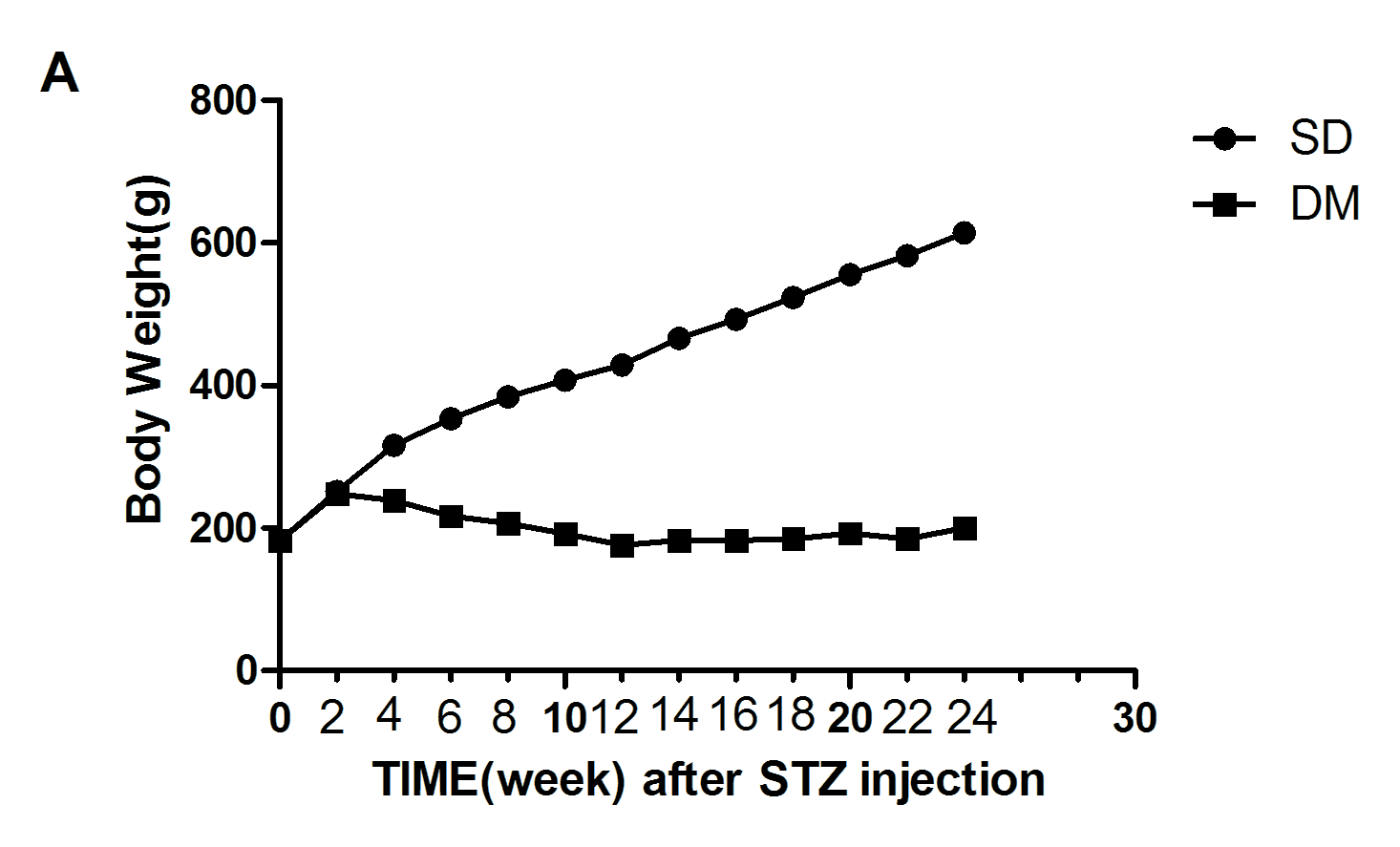

Supplement: S2 Fig — SD: the normal Sprague-Dawley rats; DM: diabetic Sprague Dawley rats. (TIF) [file pone.0185795.s002.tif]
